# Secretion of heat shock ‐60, ‐70 kD protein, IL‐1β and TNFα levels in serum of a term normal pregnancy and patients with pre‐eclampsia development

**DOI:** 10.1111/jcmm.13824

**Published:** 2018-08-22

**Authors:** María C. Álvarez‐Cabrera, Edgar Barrientos‐Galeana, Asyadette Barrera‐García, Mauricio Osorio‐Caballero, Jesús F. Acevedo, Oscar Flores‐Herrera, Néstor F. Díaz, Anayansí Molina‐Hernández, Guadalupe García‐López, Héctor Flores‐Herrera

**Affiliations:** ^1^ Departamento de Inmunobioquímica Instituto Nacional de Perinatología “Isidro Espinosa de los Reyes” (INPerIER) Ciudad de México México; ^2^ Departamento de Tococirugía INPerIER Ciudad de México México; ^3^ Departamento de Salud Sexual y Reproductiva INPerIER Ciudad de México México; ^4^ Department of Obstetrics and Gynecology University of Texas Southwestern Medical Center Dallas Texas; ^5^ Departamento de Bioquímica, Facultad de Medicina Universidad Nacional Autónoma de México Ciudad de México México; ^6^ Departamento de Fisiología y Desarrollo Celular INPerIER Ciudad de México México

**Keywords:** inflammatory cytokine, heat‐shock proteins, pre‐eclampsia, pregnancy

## Abstract

The extracellular heat shock proteins (eHsp) family act as molecular chaperones regulating folding, transporting protein and are associated with immune modulation in different physiological and pathological processes. They have been localized in different gestational tissues and their concentration in amniotic fluid and serum has been determined. In the present study, we proposed to determine the concentration of eHsp‐60, ‐70, IL‐1β and TNFα in the serum of pregnant patients with 34 weeks of gestation with and without clinical evidences of preeclampsia (PE). Our results indicate significant increase of these markers in patients with PE with respect to healthy pregnant patients without active labor. Finally, the concentration of eHsp‐60 and ‐70 correlated positively with the hepatic dysfunction markers uric acid, lactate dehydrogenase (LDH), glutamic oxaloacetic transaminase (GOT) glutamic pyruvic transaminase (GPT), and inflammatory IL‐1β and TNFα response. In conclusion, our results demonstrate a strong associated between Hsp and marker of hepatic dysfunction.

## 1 INTRODUCTION

Pre‐eclampsia (PE) is one major obstetrical hypertensive disorder affecting the development of pregnancy, is associated with maternal‐foetal morbidity and mortality and intrauterine growth restriction^1^ and complicates 8.0% of pregnancies that are treated in the clinical.^2^ Although the aetiology of PE is uncertain, the main event is attributed with the inadequate syncytiotrophoblast migration and remodelling of maternal spiral arteries results in a deficient maternal blood supply to the placenta, endothelial dysfunction, and systemic inflammatory cytokines.^3^ Within this endothelial dysfunction environment provoked by an inadequate syncytiotrophoblast remodelling have been detected extracellular heat‐shock proteins (*e*Hsp).

The *e*Hsp are highly conserved molecules that regulate cellular homoeostasis, proliferation and differentiation of the immunological cell.^4^ The *e*Hsp can be released to extracellular space in response to cellular stress by non‐classical protein transport mechanisms. Molvarec et al^5^ detected in peripheral circulation the 70‐kD heat shock protein in normal pregnant women. Several studies have demonstrated the secretion of Hsp in serum of healthy pregnant woman and increases in intra‐amniotic infection^6^ and pregnant women with PE.^7^ Here, we determine in maternal serum of pregnancy the concentration of *e*Hsp‐60, ‐70, IL‐1β and TNFα in (a) 34‐weeks of pregnancy; (b) term in labour; (c) pregnant women with PE; and (d) we determine the correlation between the *e*Hsp‐60 and ‐70 concentrations with hepatic dysfunction markers.

## 2 MATERIALS AND METHODS

### 2.1 Patients

This study was reviewed and approved by the National Institute of Perinatology Ethics and Research Committees (registration number 212250‐3210091). All patients were explained the purpose of the study, and informed consent was obtained. This study included 140 pregnant patients with no obstetric complications and no prior history of PE. Controls group (n = 78) was divided into two categories: patients with 34‐weeks of pregnancy (n = 28; the same gestational age as the group of PE) and term in labour (n = 50; with gestational age to term ≥37) defined as dilation of cervical canal (≥4 cm) and uterine contraction sustained. Patients who development clinical data of severe PE that came to the emergency unit (n = 62) was defined according to the American College of Obstetricians and Gynecologists guidelines.^8^ PE patients were included in the study previous of the therapeutic treatment. In all cases, blood samples of all patients were taken in only one occasion. The serum was obtained from 5 millilitres of peripheral maternal blood samples and stored at −80°C for the quantification of the *e*Hsp‐60, ‐70, IL1‐β and TNFα by specific immunoassay ELISA.

### 2.2 Biochemical assays

Commercial ELISA kits were used to measure concentration of *e*Hsp‐60, ‐70, IL1β and TNFα (R&D Systems, Minneapolis, MN, USA). Standard curve was development from 1.25 to 80 ng/mL, 312.5 to 20,000 pg/mL, 4.0 to 260 pg/mL and 15.0 to 960 pg/mL, respectively, according to commercial manufacturer instructions and has been previously reported by our research group,^9^ with a sensitivity of 0.70; 150.0; 2.0 and 5.0 pg/mL, respectively. The values obtained were expressed as pg/mL. Uric acid, creatinine, lactate dehydrogenase (LDH), glutamic oxaloacetic transaminase (GOT) and glutamic pyruvic transaminase (GPT) were measured in a VITROS^®^ 4600 Chemistry System (Ortho Clinical Diagnostics, Raritan, NJ) using specific kits. These measurements were determined in the central laboratory of INPerIER.

### 2.3 Statistical analysis

Proportional data were analysed using one‐way ANOVA, and significant difference between groups were determined by Tukey′s test. The Spearman rank correlation (*r*) between *e*Hsp and IL‐1β, TNFα and clinical parameters were determined. All the assays were independently replicated at least three times, and the data were presented as mean ± SEM. Significant difference was accepted at *P* value ≤0.05.

## 3 RESULTS AND DISCUSSION

Abnormal morphogenesis of the placenta is the main cause for the development of PE, which is associated with spatial and temporal secretion of various markers of endothelial damage and anti‐angiogenic factors. It has been demonstrated that molecules secreted into the cell as “alarm system” are *e*Hsp which modulate different cellular process and pathological conditions.^10^

**Figure 1:**
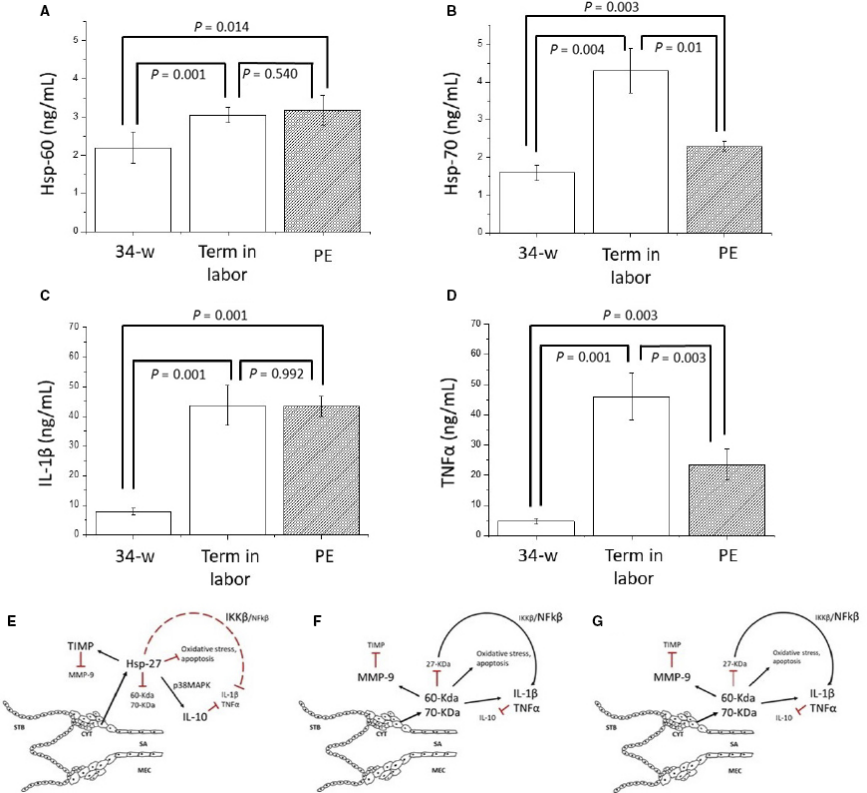
Concentration of eHsp and cytokine proinflammatory in serum of pregnant patients. Secretion profile of eHsp‐60 (A), ‐70 (B), IL‐1β (C), TNFα (D) in normal pregnancy with 34‐weeks (n = 28), term in labor (n = 50) and patients with clinical evidences of preeclampsia (PE; n = 62). The data is shown as the mean ± SEM. The statistical difference is indicated. Action model of eHsp secretion during pregnancy in normal 34‐weeks without (E), and with active labor (F) and preeclampsia condition (G). At 34‐weeks′ gestation the principal secretion is of the 27‐Kilo Daltons of heat shock protein and induced the secretion of anti‐inflammatory interleukin (IL)‐10 via intracellular p38MAPK pathways which reduce the secretion of proteins associated with oxidative stress, apoptosis, and the inflammatory response^10^ (E); however, this secretion profile changes when the labor is activated and eHsp‐60, ‐70, IL‐1β and TNFαincrease (F). In the development of PE the same events occur as in labor at term; however, this response occurs prematurely (34 weeks′ gestation, G). syncytiotrophoblast (STB), cytotrophoblast (CYT), spiral arteries (SA), maternal endothelial cells (MEC)

In this study, we found an increase significantly in the term labour group with respect to 34‐weeks′pregnancy by 1.3‐ (*P* = 0.001), 2.6‐ (*P* = 0.004), 5.5‐ (*P* = 0.001) and 9.8‐fold (*P* = 0.001) for *e*Hsp‐60, and ‐70, IL‐1β and TNFα, respectively (Figure 1). Interestingly, in patients with PE had a significant increased by 1.44‐fold (*P* = 0.014), and 1.5‐ (*P* = 0.003), 5.4‐ (*P* = 0.001) and 4.6‐fold (*P* = 0.003) for *e*Hsp‐60, and ‐70, IL‐1β and TNFα, concentrations, respectively, with respect to 34‐week pregnancy (Figure 1). Furthermore, our results showed significant correlation between Hsp‐60 and LDH (*r* = 0.620; *P* = 0.0129); GOT (*r* = 0.521; *P* = 0.0129), GPT (*r* = 0.578; *P* = 0.023), IL‐1β (*r* = 0.699; *P* = 0032) and TNFα (*r* = 0.720; *P* = 0.0034) in the PE patients (Table 1). Finally, a significant correlation coefficient between Hsp‐70 and uric acid (*r* = 0.632; *P* = 0.001), LDH (*r* = 0.769; *P* = 0.001); GOT (*r* = 0.613; *P* = 0.0023), GPT (*r* = 0.601; *P* = 0.02), IL‐1β (*r* = 0.760; *P* = 0.001) and TNFα (*r* = 0.690; *P* = 0.003) was observed in the PE patients (Table 1).

**Table 1:** Characteristics of the study population and concentration of biochemical parameters

Parameter	PE^a^ (n = 62)	CONTROL	
		34‐weeks pregnancy^b^ (n = 28)	Term in labor^c^ (n = 50)
Maternal age (y)	29.9 ± 7.3	28.3 ± 8.0	22.2 ± 6.3
BMI	28.86 ± 6.3	25.84 ± 5.5	26.3 ± 5.2
Systolic blood pressure (mm Hg)	149.9 ± 15.3	106.5 ± 9.8	109.8 ± 12.7
Diastolic blood pressure (mm Hg)	93.14 ± 9.7	67.07 ± 6.6	69.2 ± 8.9
Biochemical parameters			
Uric acid (mg/mL)	5.07 ± 1.5	4.16 ± 1.5	3.8 ± 0.72
Creatinine (mg/mL)	0.60 ± 0.26	0.54 ± 0.08	0.52 ± 0.08
LDH (UI/L)	358.43 ± 114	ND	ND
GOT (UI/mL)	23.8 ± 24.1	ND	ND
GPT (UI/mL)	22.48 ± 20.4	ND	ND
Gestational age, (wk)	34.0 ± 3.9	34.0 ± 4.4	38.0 ± 2.9

Data expresses as mean ± SD. Statistical difference (*P* < 0.05). Maternal age: (a vs c) and (b vs c); systolic blood pressure: (a vs b) and (a vs c); diastolic blood pressure: (a vs b) and (a vs c); Uric acid: (a vs b) and (a vs c).

BMI, body‐mass index; GOT, glutamic oxaliacetic transaminases; GPT, glutamic piruvic transaminases; LDH, lactate dehydrogenase; ND, no determined; PE, Pre‐eclampsia.

In normal pregnancy, syncytiotrophoblast cells invade and remodel the maternal spiral arteries and different adhesion molecules, angiogenic factors, metalloproteinases and *e*Hsp‐27 are detected. The *e*Hsp are spatially and temporally expressed in the human placenta throughout pregnancy. In the first trimesters of pregnancy is mainly of the 27‐kD protein; however, this changes to the early stages of labour (37‐weeks) reducing the expression of Hsp‐27 and increasing the 60 and 70‐kD proteins (Figure 1F).^11^

The biological significance of *e*Hsp‐27 activity during the pregnancy in the absence of cell damage suggests that these proteins are involved in cellular protection by reducing the production of molecules associated with oxidative stress, apoptosis, inflammatory response^10^ regulating trophoblast differentiation and migration^12^ (Figure 1E).

Interestingly, induce also expression of protein inhibitor α, a negative regulator of the classical nuclear transcription factor‐kappa B (NFκβ) pathway.^10^

In this study, we found that in patients with 34‐week pregnant, the secretion profile of Hsp‐70 was 1.6 ± 0.12 ng/mL (Figure 1). Similar concentrations of Hsp‐70 were reported previously by Molverac et al and Fukushima et al^5^, ^13^ they detected not significant changes in the concentration of Hsp‐70 in different stages of pregnancy without active labour.

Additionally, we demonstrated that the secretion profile concentrations increase in patients with active labour with respect to 34‐week pregnant (Figure 1). It has been shown that the profile of Hsp in labour activation increased the 70‐ and reduce the 27‐kD protein inducing contraction in the myometrium, secretion of prodegradative matrix metalloproteinase (MMP)‐2 and ‐9, nitric oxide production and inflammatory cytokines secretion.^14^

Premature intracellular activation pathways induced by the change in Hsp expression have been determined in other pregnancy pathologies.^5^, ^11^ Our results demonstrated an increase in *e*Hsp‐60 and ‐70 which correlated positively with the inflammatory response secretion (IL‐1β and TNFα) and with vascular damage (acid uric, DHL, GOT and GPT) in patients with clinical evidences of PE as compared to normal pregnancy at the same gestational age (Figure 1). Peracoli et al^15^ found that both in early and late development of PE, there was a significant increase in *e*Hsp‐70, IL‐1β and TNFα in patients with 34 weeks and PE development. Indeed, the extracellular secretion of 70‐kD protein interaction with Toll‐like receptor activated the NFkβ pathways increasing the secretion of IL‐1β and TNFα.^10^

In summary, this study demonstrated that a correlation between *e*Hsp‐60 and ‐70 with respect to hepatic and vascular damage dysfunction markers and this *e*Hsp could be used as a simple tool for the detection in the development of PE.

## CONFLICT OF INTEREST

The authors declare that they have no competing interests to disclose.

## AUTHOR CONTRIBUTIONS

MCAC, ABG and MOC identified patients with clinical evidence of PE and obtained the blood samples from the three study groups. MCAC, EBG, GGL carried out the determination of heat shock protein and inflammatory cytokine. JFA perform the correlation analysis. MCAC, OFH, NFD, AMH and HFH were involved with the study design. HFH obtained the funding and writing the manuscript. NFD and JFA discussed the manuscript in its final form. All authors read and approval the final manuscript.
